# Novel mechanism of regulation of fibrosis in kidney tumor with tuberous sclerosis

**DOI:** 10.1186/1476-4598-12-49

**Published:** 2013-05-25

**Authors:** Sitai Liang, Gabriela Cuevas, Shaza Tizani, Tiffanie Salas, Huijuan Liu, Baojie Li, Samy L Habib

**Affiliations:** 1 Geriatric Research, Education and Clinical Department, South Texas Veterans Health Care System, San Antonio, TX, USA; 2 Bio-X institutes, Key Laboratory for the Genetics of Developmental and Neuropsychiatric Disorders, Ministry of Education, Shanghai Jiao Tong University, Shanghai, China; 3 Department of Cellular and Structural Biology, University of Texas Health Science Center at San Antonio, San Antonio, TX, USA

**Keywords:** Angiomyolipoma, Fibrosis, α-SMA, YY1 and TSC

## Abstract

**Background:**

Deficiency in tuberin results in activation the mTOR pathway and leads to accumulation of cell matrix proteins. The mechanisms by which tuberin regulates fibrosis in kidney angiomyolipomas (AMLs) of tuberous sclerosis patients are not fully known.

**Method:**

In the present study, we investigated the potential role of tuberin/mTOR pathway in the regulation of cell fibrosis in AML cells and kidney tumor tissue from tuberous sclerosis complex (TSC) patients.

**Results:**

AML cells treated with rapamycin shows a significant decrease in mRNA and protein expression as well as in promoter transcriptional activity of alpha-smooth muscle actin (α-SMA) compared to untreated cells. In addition, cells treated with rapamycin significantly decreased the protein expression of the transcription factor YY1. Rapamycin treatment also results in the redistribution of YY1 from the nucleus to cytoplasm in AML cells. Moreover, cells treated with rapamycin resulted in a significant reduce of binding of YY1 to the αSMA promoter element in nuclear extracts of AML cells. Kidney angiomyolipoma tissues from TSC patients showed lower levels of tuberin and higher levels of phospho-p70S6K that resulted in higher levels of mRNA and protein of αSMA expression compared to control kidney tissues. In addition, most of the α-SMA staining was identified in the smooth muscle cells of AML tissues. YY1 was also significantly increased in tumor tissue of AMLs compared to control kidney tissue suggesting that YY1 plays a major role in the regulation of αSMA.

**Conclusions:**

These data comprise the first report to provide one mechanism whereby rapamycin might inhibit the cell fibrosis in kidney tumor of TSC patients.

## Background

Tuberous Sclerosis complex (TSC) is a genetic disorder that causes tumors to form in many organs. Loss of heterozygosity (LOH) at the *TSC1* or *TSC2* loci has been detected in *TSC*-associated hamartomas and renal cell carcinoma (RCC) as well as in sporadic tumors of non-TSC patients [1,2]. Kidney cancer development is rare in TSC, occurring in only 2–3% of all patients [3-5]. Multicentric angiomyolipomas are much more common in patients with *TSC* than RCCs, but may, nonetheless, have similar underlying genetic basis at early steps in their genesis and/or progression, specifically in the setting of tuberin deficiency [6]. Angiomyolipomas (AMLs) are benign kidney tumors composed of smooth muscle, fat, and vessel cells. Renal angiomyolipomas tend to be larger, bilateral, multifocal and present at a younger age compared with sporadic forms [7]. Angiomyolipomas most commonly occur in the kidneys and, although often asymptomatic, may enlarge and bleed leading to hemorrhage and renal impairment [8-10]. The lesions are usually benign although rare malignant cases have been described, often consisting of only the smooth muscle component [10].

The *TSC2* gene product, tuberin, is a tumor suppressor protein whose absence or inactivation is associated with several defects such as abnormal cellular migration, proliferation, and differentiation [11-13]. Tuberin expression was initially induced following acute renal injury, suggesting that the *TSC2* may function as an acute-phase response gene, limiting the proliferative response after injury [14]. The smooth muscle cell component of angiomyolipoma is thought to derive from a precursor termed the perivascular epithelioid cell (PEC) [15]. PEC-derived lesions, which include lymphangioleiomyomatosis (LAM), a cystic lung disease, are characterized by the dual expression of smooth muscle and melanocytic features [16]. Angiomyolipoma is rare in the general population but is present in 80% of patients with TSC, an autosomal dominant tumor suppressor syndrome characterized by multiple hamartomas in the central nervous system, skin and other organs [17]. Although TSC occurs equally in men and women, angiomyolipoma is more common and larger in women and is thought to be estrogen dependent. *In vitro*, the growth of cells derived from angiomyolipoma can be enhanced by estrogen [18]. Thus, it is likely that although LAM and angiomyolipoma result from the loss of tuberin or hamartin. Unlike other TSC related hamartomas, their growth is promoted by estrogen.

The mammalian target of rapamycin (mTOR) serves a critical role in regulating the translational machinery that affects growth, proliferation, and differentiation, all of which are abnormally manifested in TSC lesions [19,20]. These lesions are associated with accumulation of fibrotic proteins but the mechanisms by which tuberin regulates these proteins has not been explored. In the current study, we examined the role of tuberin/mTOR in regulating α-SMA in TSC patients. Here, we report for the first time that tuberin/mTOR pathways regulate α-SMA through redistribution the transcription factor YY1 from the cytoplasm to the nucleus in AML cells. In addition, binding of YY1 to α-SMA promoter may provide a mechanism for enhancing the expression α-SMA to increase cell fibrosis in kidney angiomyolipoma of TSC patients.

## Results

### Inhibition of mTOR decreases protein and mRNA expression of αSMA

**Figure 1:**
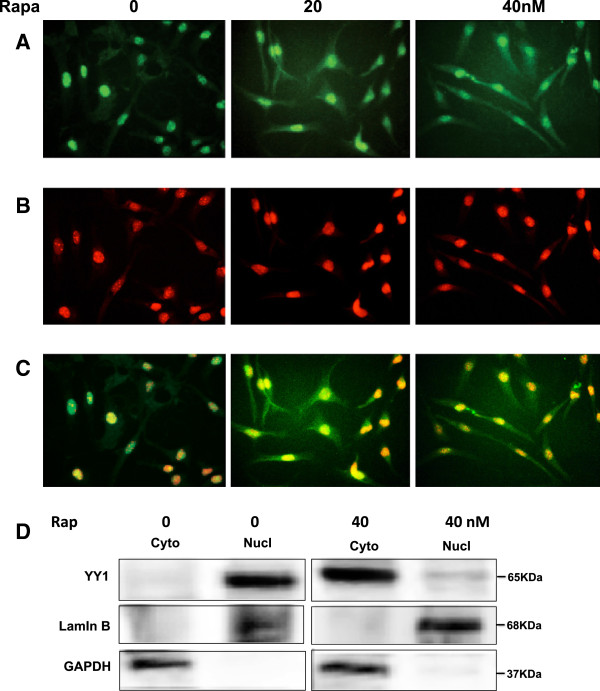
**Rapamycin decreases of αSMA in AML cells.** AML cells were treated with different concentrations of rapamycin (0-40 nM) for 24 h. Western blot analysis was performed in cell lysates using αSMA antibody. (**A**) Cells treated with 40 nM of rapamycin showed significant decrease in protein expression of αSMA. Inhibition of mTOR decreases mRNA of αSMA in AML cells. RNA from untreated and rapamycin treated AML cells were extracted and subjected to RT-PCR analysis. (**B**) mRNA of αSMA products were separated on agarose gel electrophoresis and visualized by ethidium bromide staining under UV light. (**A**) Cells treated with 40 nM of rapamycin showed also significant decrease in mRNA of αSMA. GAPDH was used as a loading control. Significant difference from untreated cells is indicated by **P < 0.01 and *P < 0.05. Rapamycin significantly decreased YY1 protein expression and αSMA promoter transcriptional activity in AML cells. AML cells were treated with different concentrations of rapamycin (0-40 nM) for 24 h. (**C**) Protein from untreated and rapamycin treated AML cells were extracted and subjected to Western blot analysis. Treated AML cells with rapamycin (0-40 nM) for 24 results in significant decreased in YY1 protein expression (**D**) A reporter plasmid containing the αSMA promoter driving expression of the luciferase and a control *Renilla* reporter gene were co-transfected into the cells using LipofectAMINE Plus Reagent^TM^. After treatment with different concentrations of rapamycin for 24 h. Luciferase activity was determined using the Luciferase Reporter Assay System by a luminometer and normalized by *Renilla* reporter activity. Histograms represent means ± SE (n = 6). Significant difference from cells treated with rapamycin is indicated by *P < 0.01.

To determine the effect of mTOR inhibition on αSMA, AML cells were treated with different concentrations of rapamycin (0-40 nM) for 24 h. Protein and RNA from untreated and rapamycin treated cells were extracted and subjected to Western blot and RT-PCR analysis. Cells treated with rapamycin (40 nM) showed significant decrease in mRNA of αSMA products compared to untreated cells or cells treated with low concentrations of rapamycin (20nM) (Figure 1A). In addition, significant decreased in αSMA protein expression showed in cells treated with same concentration of rapamycin (Figure 1B). These data suggests that inhibition of mTOR by rapamycin is sufficient to downregulate the αSMA at transcription and translation levels.

### Rapamycin significantly decreased YY1 protein expression and αSMA promoter activity

To determine whether decreases in mRNA and protein expression were reflected in changes in αSMA promoter activity through YY1, AML cells were transfected with αSMA promoter luciferase construct. Cells treated with different concentrations of rapamycin (0-40 nM) for 24 h showed a significant decrease in YY1 protein expression by Western blot analysis (Figure 1C). YY1 is a potential transcription factor that regulates αSMA. In the next experiment we have tested the effect of rapamycin treatment on regulation of the promoter activity of αSMA in AML cells. A reporter plasmid containing the αSMA promoter driving expression of the luciferase and a control *Renilla* reporter gene were co-transfected into AML cells. Cells were treated with different concentrations of rapamycin (0-40 nM) for 24 h then luciferase activity was determined using the Luciferase Reporter Assay System by a luminometer and normalized by *Renilla* reporter activity. Data in Figure 1D showed that rapamycin treatment resulted in a significant decrease in αSMA promoter activity compared to untreated cells suggesting the role of mTOR in transcriptional regulation of αSMA.

### Inhibition of mTOR results in redistribution of YY1 from the nucleus to cytoplasm in AML cells

**Figure 2:**
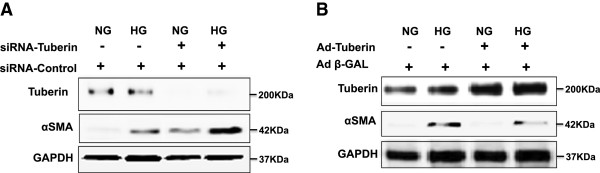
**Rapamycin treatment results in the redistribution of YY1 from the nucleus to cytoplasm in AML cells.** Immunofluorescene staining was used to detect the localization of YY1 in AML cells treated with rapamycin (0–40 nM) for 24 hours. (**A**) FITC green signals for YY1 were detected using a filter with excitation range of 488 nm and (**B**) propidium iodide red signals for nuclear DNA using a filter with excitation at 535 nm. (**C**) Overlay of YY1 and DNA staining, demonstrating nuclear localization of YY1 in AML cells treated with rapamycin. To show staining specificity, control cells were stained without primary antibody. (**D**) Rapamycin treatment significantly decreased YY1 in the nuclear fraction. Lamin B was used as a nuclear marker and GAPDH as cytoplasmic marker.

Immunofluorescene staining was used to detect the localization of YY1 in AML cells treated with rapamycin (0–40 nM) for 24 hours. Overlay staining of YY1 and DNA staining, demonstrating nuclear localization of YY1 in non treated AML cells while cytoplasmic staining of YY1 was detected in treated cells with rapamycin (Figure 2A-C). We have confirmed the redistribution of YY1 by Western blot analysis in cytoplasmic and nuclear fractions of untreated and treated AML cells with 40 nm rapamycin (Figure 2D). These data suggested that treatment with rapamycin increases the cytoplasmic redistribution of YY1 that may results in decreased αSMA in nucleus. However, redistribution of YY1 localization by rapamycin indicating that activation of the mTOR pathway plays an important role in the activation of fibrosis signaling in AML cells.

### Tuberin regulates αSMA

**Figure 3:**
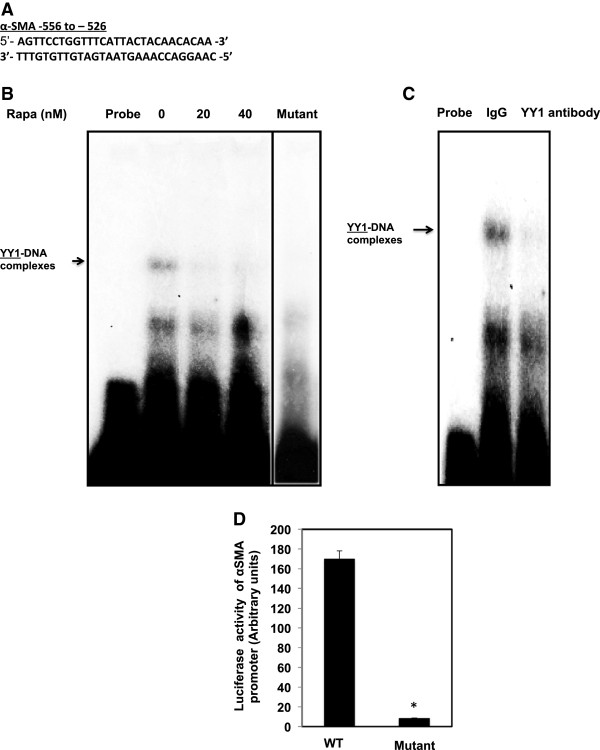
**Tuberin regulates αSMA expression in human cells.** (**A**) Immunoblot analysis shows that downregulation of tuberin with siRNA directed against TSC2 results in an increased in αSMA protein expression in human renal embryonic epithelial cells (HEK293) in cells grown in normal glucose or pretreated with high glucose (25 mM) for 24 h. (**B**) Overexpression of tuberin decreases αSMA expression. HEK293 cells were infected with adenovirus 6.01 expressing tuberin complementary DNA. An adenovirus vector expressing protein (Ad-GAL) was used as a control. Overexpression of tuberin results in decreased αSMA protein expression in cells grown in normal glucose or pretreated with high glucose (25 mM) for 24 h. GAPDH was used as a loading control.

To determine whether tuberin regulates αSMA expression through *TSC2*, tuberin was downregulated using duplex siRNA oligonucleotide complementary to *TSC2* in human embryonic kidney (HEK293) cells. Downregulation of tuberin results in increased αSMA expression compared with cells transfected with scrambled control oligonucleotides (Figure 3A). In contrast, overexpression of tuberin in HEK293 cells using an adenovirus expressing tuberin (Ad-*TSC2*) results in decreased αSMA protein expression, indicating that tuberin is an upstream regulator of αSMA in AML cells. In addition, cells transfected with siRNA against tuberin and grown in normal glucose or treated high glucose (HG, 25 mM) for 24 h showed a significant increase in αSMA protein expression (Figure 3B). Moreover, cells infected with adenovirus expressing tuberin and treated with HG confirmed that upregulation of tuberin resulted in significant decrease in αSMA protein expression under both low and high-energy conditions (Figure 3B).

### Rapamycin treatment significantly reduces the binding of YY1 to the αSMA promoter element

**Figure 4:**
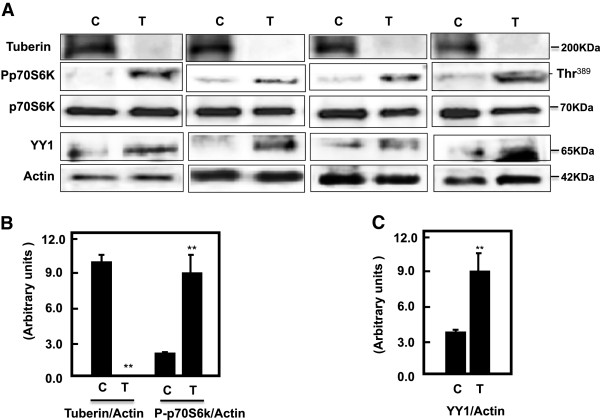
**Rapamycin treatment significantly reduces the binding of YY1 to the αSMA promoter element.** (**A**) EMSA analysis of a DNA probe corresponding to the putative YY1 binding site in the αSMA promoter. Labeled probes were incubated with nuclear extracts isolated from untreated and treated AML cells with different concentrations of rapamycin (0–40 nM). (**B**) Treatment of AML cells with 20-40 nM of rapamycin significantly reduced YY1 in the nuclear extracts compared to untreated cells and cells. Mutant of YY1 oligonucleotide in TTT to AAA base substitutions showed no binding to YY1 promoter-specific DNA complexes (Lane 5 was moved from its original position on the gel). (**C**) The specificity of binding of the DNA/protein complex to YY1 was demonstrated by adding YY1 antibody to the reaction mixture. Including the YY1 antibody in the reaction results in markedly reduced the specific DNA/protein complex. (**D**) A reporter plasmid of αSMA promoter driving expression of the luciferase contained wild type or mutant of YY1 was transfected into AML cells. Control *Renilla* reporter gene was co-transfected into the cells using LipofectAMINE Plus Reagent^TM^. Luciferase activity was determined using the Luciferase Reporter Assay System by a luminometer and normalized by *Renilla* reporter activity. Histograms represent means ± SE (n = 6). Significant difference from cells transfected with wild type YY1 is indicated by *P < 0.01.

To further investigate the mechanism by which inhibition of mTOR affect the binding of YY1 to DNA-binding site in the αSMA promoter, EMSA was performed using nuclear extracts of AML cells. Intact 28-bp double-strand end-labeled oligonucleotides containing the sites spanning the region −556 to −526 of the YY1 that binds to the αSMA promoter was used as a DNA probe (Figure 4A). Labeled probe was incubated with nuclear extracts isolated from untreated or treated rapamycin AML cells. Treatment of AML cells with 20-40 nM rapamycin significantly reduced YY1 in the nuclear extracts compared to untreated cells (Figure 4B). In addition, incubation of nuclear extracts with mutant of YY1 oligonucleotide in TTT to AAA base substitutions showed no binding to YY1 promoter-specific DNA complexes. To confirm the specificity of the protein-DNA interaction, the cell extracts were also pre-incubated with an antibody recognizing YY1 (Active Motif, Carlsbad, CA) or with a nonspecific antibody. The DNA-protein complexes were significantly abolished in the presence of the YY1 antibody, but not by IgG, indicating that YY1 is indeed a component of these complexes (Figure 4C). These data suggest that inhibition of mTOR results in regulation the binding of YY1 to αSMA promoter to block cell fibrosis in AML cells.

### Mutation of YY1 reduces αSMA promoter activity

αSMA plasmid constructs contained wild type (WT) and mutant of YY1 driving expression of the luciferase were transfected into AML cells. A control *Renilla* reporter gene was co-transfected into AML cells. Luciferase activity was determined using the Luciferase Reporter Assay System by a luminometer and normalized by *Renilla* reporter activity. Data in Figure 4D showed that mutation in YY1 significantly decreased αSMA promoter activity and confirmed the role of YY1 in the regulation of transcriptional activity of αSMA.

### Deficiency in tuberin and activation of mTOR result in increased YY1 in tumor kidney tissue of TSC patients

**Figure 5:**
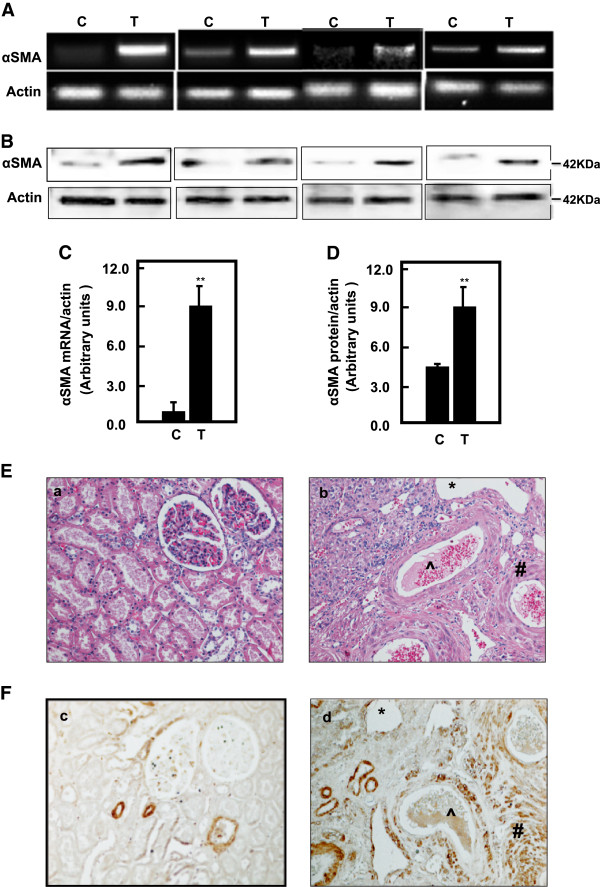
**Deficiency of tuberin and activation of mTOR result in increased YY1 protein expression in tumor kidney tissue of TSC patients.** (**A**) Representative Immunoblot analysis showed significant decreased in tuberin and increased in phospho-p70S6K as well as YY1 protein expression in tumor kidney (T) from patients with tuberous sclerosis compared to normal kidney tissues. Actin was used as loading control. (**B** &**C**’) Histograms represent means ± SE (n = 6). Significant difference from control is indicated by **P < 0.01.

Tuberin and phosho-p70S6K as well as total p70S6K protein expression were measured in normal kidney (C) and tumor kidney (T) from patients with tuberous sclerosis by Western blot analysis. Data in Figure 5A & B showed significant decreased in tuberin expression was associated with increased in mTOR activity that measured by phosphorylation of p70S6K at Thr^389^ confirming that deficiency in of tuberin activates mTOR in kidney of TSC patients. Deficiency in tuberin and increased mTOR activity result in increased the protein expression of YY1 in tumor kidney tissue of patients with TSC (Figure 5A & C).

### Upregulation of YY1 significantly increased in αSMA in tumor kidney tissues of TSC patient

**Figure 6:**
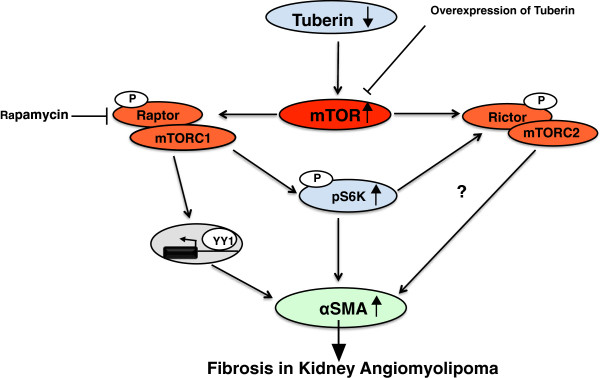
**Upregulation of YY1 results in significant increased in mRNA and protein expression of αSMA in tumor kidney tissues of TSC patients.** Representative RTPCR of mRNA (**A**) and immunoblot protein expression (**B**) of αSMA in normal kidney and tumor kidney tissues from patients with tuberous sclerosis. Actin was used as loading control. (**C**’ &**D**) Histograms represent means ± SE (n = 6). Significant difference from control is indicated by **P < 0.01. (**E**) H&E staining shows (**a**) a normal tubular and glomerular structure in control kidney tissue and (**b**) *fat, ^vessel and # smooth muscle cells types in kidney angiomyolipoma tissue of TSC patients. (**F**) Kidney sections were stained with αSMA followed by horseradish peroxidase staining. (**c**) Control of kidney shows a few cells stained with αSMA while (**d**) most of blood vessel and smooth muscle cells were stained in kidney tumor tissue. Control sections in both procedures were incubated without primary antibody.

Protein and mRNA expression of αSMA in normal and tumor kidney tissues from patients with tuberous sclerosis were analyzed by Western blot and RT-PCR, respectively. Data in Figure 6A & C showed a significantly increase in mRNA of αSMA in tumor kidney tissue compared to normal tissues. In addition, protein expression of αSMA was significantly increased in tumor kidney tissue compared to normal tissues (Figure 6B & D). These data indicate that deficiency in tuberin resulted in upregulation αSMA protein expression and increase cell fibrosis through increased YY1 expression in kidney angiomyolipomas of TSC patients. Data in Figure 6E (a & b) showed H&E staining of (a) normal tubular and glomerular structure in control kidney tissue and (b) fat, vessel and smooth muscle cells types in kidney angiomyolipoma tissue of TSC patients. Kidney sections from normal and tumor of TSC patients were stained with αSMA followed by horseradish peroxidase staining. Data in Figure 6F (c & d) showed a few cells stained with αSMA in normal kidney tissue while (d) most of blood vessel and smooth muscle cells were stained with αSMA in kidney tumor tissue.

## Discussion

This study provides the first evidence that tuberin regulates the expression and promoter activity of the cell fibrosis protein αSMA and that tuberin exerts this effect at least partially through the transcription factor, YY1. The data demonstrate that tuberin deficiency in AML cells is associated with upregulation of αSMA protein and mRNA, as well as with marked increase of αSMA promoter activity. Downregulation of tuberin in HEK293 renal cells is associated with increased protein expression of αSMA. These data indicate a novel role for tuberin in the regulation of cell fibrosis protein and provide a potential mechanism by which *TSC2* mutations and tuberin deficiency predispose to the genesis and progression of fibrosis in kidney angiomyolipomas of TSC patients. Several approaches were utilized to conclusively demonstrate that tuberin/mTOR regulates αSMA. First, inhibition of mTOR by rapamycin was associated with decrease in αSMA mRNA and protein in AML cells. Second, inhibition of mTOR by rapamycin inhibits the promoter activity of αSMA in AML through decreased the protein expression of YY1. Third, rapamycin treatment resulted in redistribution of YY1 from the nucleus to cytoplasm in AML cells. Fourth, downregulation of tuberin in renal epithelial cells by siRNA against TSC2 was associated with increase in αSMA protein. Upregulation of tuberin by introduction of *TSC2* cDNA into the tuberin-deficient cells into HEK293 cells results in significant decreased in αSMA protein expression. In addition, Inhibition of mTOR results in regulation the binding of YY1 to αSMA promoter to block fibrosis in AML cells. Mutation in YY1 blocked the binding of YY1 to αSMA promoter and resulted in significant decrease of αSMA promoter activity. Moreover, higher levels of mRNA and protein of αSMA were detected in kidney angiomyolipoma tissue compared to control kidney tissues. Furthermore, tumor tissue showed higher protein expression of YY1 compared to control kidney tissue suggesting that YY1 plays a major role in the regulation of fibrosis in kidney angiomyolipoma tissue.

In general, fibrosis results from activation and differentiation of fibroblasts, which overexpress extracellular matrix proteins including αSMA. The decrease in αSMA mRNA in AML cells suggests that decreased transcription is one potential mechanism responsible for downregulation of αSMA protein. YY1 has recently been shown to regulate the transcription of αSMA in lung fibroblast cells [21]. YY1 binds to the αSMA promoter and activates αSMA transcription. The YY1 binds to αSMA promoter in the region between −556 and −526, which are important for binding of YY1 to the consensus sequence of αSMA promoter. Indeed, using EMSA, we found that YY1 binding was significantly decreased in AML cells treated with rapamycin. The specificity of the DNA protein complex was confirmed by demonstrating significant decrease in the binding of protein to DNA in the presence of anti-YY1antibody. Such antibodies did significantly block nuclear proteins from binding to αSMA promoter sites (Figure 3C). In addition, mutation in YY1 significantly decreased αSMA promoter activity in AML cells. Transcriptional activity using a heterologous αSMA-luciferase promoter in AML cells was also decreased in cells treated with rapamycin, clearly demonstrating that tuberin/mTOR regulates α-SMA through induction of its transcription. Collectively, the data indicate the major role of YY1 in the regulation of αSMA and fibrosis of kidney angiomyolipomas of TSC patients.

Our data showed that deficiency in tuberin and activation of mTOR resulted in upregulation of YY1 in tumor kidney tissue of TSC patients. The increase in mRNA and protein expression of αSMA was associated with increase of YY1 protein expression in tumor kidney tissues. In addition, the immunostaining data confirmed that most of blood vessel and smooth muscle cells were stained with αSMA in kidney tumor tissue. These data suggest that deficiency in tuberin resulted in upregulation αSMA and increased cell fibrosis through increased YY1 protein expression in kidney angiomyolipomas of TSC patients. Because YY1 can directly bind to the promoter regions of α-SMA gene [21] and is required for its expression, we suggest that YY1 directly regulates a-SMA that is involved in fibroblast activation and myofibroblast differentiation in kidney angiomyolipomas. Thus, decreasing YY1 expression may attenuate fibrotic responses by multiple mechanisms, in keeping with a growing role for YY1 in cell survival in response to injury [22,23]. In addition to direct effects in fibroblasts, decreasing YY1 expression by rapamycin may inhibit early inflammation during injury and repair responses, and may help to protect against kidney fibrosis by this mechanism. In support of this idea, we showed that inhibition of mTOR by rapamycin decreased YY1 protein expression and significantly decreased the promoter activity of αSMA in AML cells. YY1 is generally considered a constitutively expressed nuclear phosphoprotein, but there is growing evidence that YY1 expression can be dynamically regulated by chemotherapy. Our data in Figure 2 showed the redistribution of YY1 from nucleus to cytoplasm in AML cells treated with rapamycin supporting this idea. In addition, case study showed that treatment of TSC patients with low dose of rapamycin reduced the facial angiofibromas, renal AML volume, improved of blood pressure control and absent bleeding over 12 months of treatment suggesting the role of rapamycin as an anti-fibrotic and anti-proliferative drug [24].

## Conclusions

**Figure 7:**
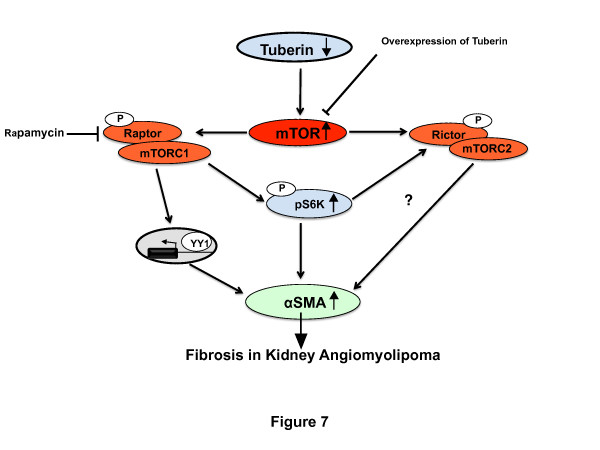
**Proposed model of regulation of fibrosis in kidney angiomyolipoma of tuberous sclerosis patients.** Deficiency in tuberin and activation of mTORC1 increased the accumulation of αSMA through upregulation of YY1 to fibrosis proteins in kidney angiomyolipoma of TSC patients.

In conclusions, deficiency in tuberin is associated with increased expression of αSMA in AML cells as well as in kidney tumor of patients with TSC. Treatment of AML cells with mTOR inhibitor significantly decreased the accumulation of αSMA, suggesting that tuberin plays a significant role in protecting the cells from fibrosis. In addition, deficiency of tuberin is sufficient to increase accumulation of αSMA, with consequent increased the transcriptional activity of αSMA through upregulation of YY1 (Figure 7). The mechanisms by which tuberin deficiency regulates kidney fibrosis in angiomyolipomas requires further study, including investigating the potential of YY1 that directly regulates several genes involved in fibrosis.

## Materials and methods

### Human tissue

Kidney angiomyolipoma tissue from TSC patients with renal angiomyolipoma (total of 6) and unrelated healthy people (total of 6) were obtained from the Brain and Tissue Bank for Development Disorders (University of Maryland, Baltimore, Maryland, USA). The study has been approved by the Institutional Review Board of The University of Texas Health Science Center at San Antonio, TX.

### Cell culture

Angiomyolipoma cells derived from kidney human of TSC patient were generously provided by Dr. Elizabeth Henske (Harvard Medical School, MA). The cells were grown in DMEM supplemented with 10% FBS. Human embryonic kidney epithelial cells (HEK293) were obtained from American Type Culture Collection (Manassas, VA) and maintained in DMEM with 10% FBS. All cell lines were grown at 37°C in a humidified atmosphere of 5% CO_2_.

### Overexpression of tuberin

The human embryonic kidney (HEK293) cells were grown to 30–50% confluence, made quiescent by serum deprivation for 24 h and then infected at room temperature for 1 h with adenovirus 6.01 carrying the Tsc2 gene [25]. Viral stocks were prepared and tittered using the serial dilution technique as described in the Adeno-X Expression Systems User Manual (Clontech Laboratories). Infection of cells with 20 multiplicity of infection (MOI) showed appreciable expression of TSC2 protein. An adenovirus vector expressing protein (Adβ-GAL) was used as a control. The cells were then incubated for 48 h at 37°C in a humidified atmosphere of 5% CO_2_. Cells were washed twice with PBS buffer and western blot analysis was performed on the cell extracts as above using tuberin and αSMA antibodies.

### siRNA experiments

HEK293 cells were grown in six-well plates. Prior to transfection, cells (30–50% confluent) were washed with PBS and media was replaced with 800 μl of OPTI-MEM (Invitrogen, Carlsbad, CA). In parallel, 4 μl of oligofectamine (Invitrogen, NY) were combined with 11 μl of OPTI-MEM I and incubated at room temperature for 10 min. SMART selected small interfering RNA (siRNA) duplexes of TSC2 with ‘UU’ overhangs and 5′ phosphate on the antisense strand. The siRNA specific for TSC2 was a mixture of four pooled duplexes. The TSC2 siRNA kit was obtained from Dharmacon/Upstate, (Lake Placid, NY). According to the manufacturer, these siRNA efficiently blocks tuberin expression by 50–70%. The indicated duplex of 1.5 μg were diluted into 180 μl of OPTI-MEM I, added to the Oligofectamine/OPTI-MEM I mixture and incubated at room temperature for 20 min. The siRNA complexes were then added to the cells. After incubation for 3–4 h in a 5% CO_2_ incubator, 1 ml of fresh medium was added to a final serum concentration of 10%. Forty-eight hours after transfection, cells were harvested for western blot analysis. The control construct used in parallel experiments contains four, pooled, non-specific siRNA duplexes provided by with the kit [26].

### Western analysis

Homogenates of kidney cortex or cell lysates were prepared as described previously [27]. Protein concentrations were determined with the Bradford assay using bovine serum albumin as a standard [28]. Western blot analysis was performed as described previously [29]. Tuberin, phospho-p70S6K, and p70S6K antibodies were from Cell Signaling (Boston, MA); GADPH and antibody was obtained from Santa Cruz Biotechnology (Santa Cruz, CA) and Actin antibody from Calbiochem (Billerica, MA). αSMA rabbit antibody was from Abcam (Cambridge, MA). An enhanced chemiluminescence kit (Amersham, NJ) was used to identify protein expression. Expression of each protein was quantified by densitometry using National Institutes of Health image 1.62 software and normalized to a loading control.

### Electrophoretic mobility shift assay

Nuclear proteins were extracted from AML cells as described previously [26]. The protein concentration of the nuclear extracts was determined using Bradford method [28]. Electrophoretic mobility shift assay (EMSA)-binding reactions were incubated in a 20 μl final volume for 20 min at room temperature containing 5 μg of the nuclear extract, 20–30 fmol of the 5′ end-labeled double-stranded 21 bp oligonucleotide: 5’- AGTTCCTGGTTTCATTACTACAACACAA −3, covering the region of the αSMA promoter from 556 to 526 (control), and 1 μl of poly (dI-dC). Mutant oligonucleotide was designed as following: 5’- AGTTCCTGG**AAA**CATTACTACAACACAA −3. The super shift assays were performed by pre-incubating nuclear extracts with 1 μg of YY1 antibody (Santa Cruz Biotechnology) into the reaction. In addition, labeled YY1 oligonucleotide at 556 to 526 (nt)- was incubated with nuclear extracts of AML cells. The reaction was carried out at room temperature for 30 min prior to adding the radiolabelled probe. The complexes were resolved using a 5% non-denaturing polyacrylamide gel. The gels were dried and exposed overnight at −70°C.

### Immunofluorescence staining of YY1

A double fluorescence labeling method was used as described previously with minor modifications [27]. AML cells in chamber slides were serum-starved for overnight then treated with different concentrations of rapamycin (0-40 nM) for 24 hours. The cells were washed with PBS, fixed, and incubated with rabbit antibody against YY1 (Cell Signaling Technology, MA), followed by secondary anti-rabbit IgG conjugated with FITC. The cells were reacted with Vectashield Mounting Medium with Propedium Iodide (PI) (Vector Laboratories). In this assay, DNA was labeled with PI, and YY1 was identified by the primary monoclonal antibody FITC green signals. FITC green signals for YY1 were detected using a filter with excitation range 450–490 nm and propidium iodide red signals for nuclear DNA using a filter with excitation at 535 nm. FITC and propidium iodide were detected using an Olympus Research microscope equipped for epifluorescence with excitation and band pass filters. To show staining specificity, control cells were stained without primary antibody.

### Immunoperoxidase staining of αSMA

Detection of αSMA was performed on paraffin sections of normal and tumor kidney by immunoperoxidase histochemical staining [30]. Kidney sections underwent a protease digestion step before they were incubated with rabbit anti-αSMA antibody (Abcam, Cambridge, MA) for 30 min then washed twice with PBS. Sections were then incubated with horseradish peroxidase labeled anti-rabbit antibody for 30 min. The horseradish peroxidase was developed with diaminobenzidine tetrahydrochloride and hydrogen peroxide in PBS. Control sections in both procedures were incubated without primary antibody.

### Cell lysates fractionation

Cytoplasmic and nuclear protein fractions were extracted from the cell lysates using a nuclear and cytoplasmic fractionation kit (Pierce, Rockford, IL). The protein concentration of the nuclear extracts was determined using the Bradford method [28].

### Quantitation of mRNA by RT-PCR

RNA was isolated from cells treated with rapamycin (0-40 nM) using RNA Isolation Solvent (Tel-Test, Newark, NJ). In addition, RNA was extracted from kidney tissue of control and tumors samples using RNeasy Mini kit (Qiagen, Valencia, CA). RNA was quantitated by spectrophotometery at 260 nm, and its integrity tested by formaldehyde/agarose gel electrophoresis. RTPCR was performed as previously described using the primers (5’-3-ACGCTGGTCACCGTGGCGGC/5’- TTGCCGCTCTTCTTGCCGCC) for αSMA. The amplified product was 356-bp long. For GAPDH, an internal control of amplification, upstream/reverse primers were 5'- GCCACCCAGAAGACTGTGGAT /5'- GAAGGCCATGCCAGTGAGCT synthesizing a 528-bp product. The PCR products were analyzed by electrophoresis on agarose gels and ethidium bromide staining. The yield was determined by densitometry and the ratio of αSMA to actin was then calculated.

### Transcriptional activity of αSMA promoter

A mouse αSMA promoter reporter plasmid was used to determine the transcriptional activity of the αSMA gene. αSMA plasmid constructs contained wild type or mutant of YY1 were generously provided by Dr. Jia Guo (University of Rochester Medical School, Rochester, New York). A *Renilla* reporter plasmid (pRL-null) was used as transfection control. Plasmids were transfected into PPTE cells using the LipofectAMINE and Plus Reagent method (Life Technologies, NY). LipofectAMINE was added to the complex of DNA and Plus reagent and incubated for 15 min at room temperature. DNA and Plus reagent-LipofectAMINE complexes were added to each well and incubated at 37°C with 5% CO_2_. After incubation for 3–4 h, 1 ml of fresh media with 20% serum was added to a final concentration of 10%. Cells were pre-treated with rapamycin (0-40 nM) for 24 h. Forty-eight hours after transfection, cells were harvested for Firefly and *Renilla* luciferase assay using Dual-Luciferase Reporter assay kit (Promega, Madison, WI). Luciferase activity was determined using the Luciferase Reporter Assay System and a Turner luminometer according to the manufacturer's instructions (Promega, Madison, WI) and normalized to *Renilla* activity.

### Statistics

Data are presented as mean ± standard error. Statistical differences were determined using ANOVA followed by Student Dunnett’s (Exp. vs. Control) test using 1 trial analysis. *P-*values less than 0.05 and 0.01 were considered statistically significant.

## Competing interests

The authors’ declare no potential conflicts of interest with respect to the research, authorship and/or publication of this article.

## Authors’ contributions

SL, ST and TS acquired data and performed the statistical analysis. GC assisted with Western blot assays. HL and BL designed some experiments and revised the manuscript. SLH conceived and designed study; analyzed and interpreted data; drafted and approved the manuscript. All authors read and approved the final manuscript.
